# Use of lentiviral pseudotypes as an alternative to reassortant or Triton X‐100‐treated wild‐type Influenza viruses in the neuraminidase inhibition enzyme‐linked lectin assay

**DOI:** 10.1111/irv.12669

**Published:** 2019-08-13

**Authors:** Fabrizio Biuso, Laura Palladino, Alessandro Manenti, Valerio Stanzani, Giulia Lapini, Jin Gao, Laura Couzens, Maryna C. Eichelberger, Emanuele Montomoli

**Affiliations:** ^1^ VisMederi s.r.l., Strada del Petriccio e Belriguardo Siena Italy; ^2^ VisMederi Research s.r.l., Strada del Petriccio e Belriguardo Siena Italy; ^3^ Center for Biologics Evaluation and Research, Food and Drug Administration Silver Spring MD USA; ^4^ Department of Molecular and Developmental Medicine University of Siena Siena Italy

**Keywords:** enzyme‐linked lectin assay, influenza, neuraminidase, pseudovirus

## Abstract

**Background:**

Formulation of neuraminidase (NA) within influenza vaccines is gaining importance in light of recent human studies. The enzyme‐linked lectin assay (ELLA) is considered a reliable assay to evaluate human anti‐NA antibodies.

**Objectives:**

To overcome interference by hemagglutinin (HA)‐specific antibodies and detect neuraminidase inhibitory (NI) antibodies only, two different sources of antigen have been studied in ELLA: reassortant viruses with a mismatched avian origin‐HA or Triton X‐100 (Tx)‐treated wild‐type viruses. Pseudotypes or pseudovirus (PV), characterized by a lentivirus core bearing human influenza NA and avian influenza HA, were investigated as an alternative source of antigen and compared to HA‐mismatched and Tx‐treated viruses, since represent a safer product to be handled.

**Methods:**

Two independent panels of sera were analyzed by ELLA to evaluate the anti‐NA response against N1 (A/California/07/2009 (H1N1pdm)) and N2 (A/Hong Kong/4801/2014 (H3N2)). The NA inhibition (NI) antibody titers measured as either the 50% end point or 50% inhibitory concentration (IC_50_) were compared for every source of antigen.

**Results:**

The ELLA assay performed well with all three sources of antigen. NI titers measured using each antigen type correlated well when reported either as end point titers or as the IC_50_.

**Conclusions:**

This study suggests that HA‐mismatched whole virus, Triton‐treated wild‐type virus or PV can be used to measure NI antibody titers of human sera, but further comparability/validation assays should be performed to assess statistical differences. The data support the use of PV as an attractive alternative source of antigen and justify further investigation to improve stability of this antigen source.

## 1 INTRODUCTION

Neuraminidase is the second most abundant glycoprotein on the influenza virus surface (17% of the overall surface) after HA and it is usually expressed at a ratio 1:4 (40‐50 NA and 160‐200 HA spikes),^1^ with exceptions.^2^, ^3^, ^4^ NA has multiple roles: (a) allow the release of newly formed virions from the surface of the infected cell, leading to viral spread, (b) enhance influenza infection by acting on glycoconjugates expressed at the cell surface,^5^ and (c) form complexes with sialic acids on the host cell surface,^6^, ^7^ particularly for H3N2 viruses.^8^ Several studies have confirmed that both inactivated and live attenuated vaccines have the capacity to induce NA‐specific antibodies.^9^, ^10^ NA inhibiting antibodies are associated with resistance against influenza,^11^, ^12^ reduced severity and duration of disease.^13^

Several different assays have been used to evaluate the antibody response to NA. The traditional NA inhibition (NI) assay determines the extent of antibody‐mediated interference with viral enzyme activity based on the measurement of sialic acid that is released from a glycosylated substrate.^14^ An assay that measures NA activity based on accessibility of galactose, the penultimate sugar of many complex carbohydrates, to peanut agglutinin, offers advantages in that it does not use hazardous chemicals and has higher throughput. This enzyme‐linked lectin assay (ELLA) developed by Lambré et al^15^ and successively adapted and optimized,^10^, ^16^, ^17^ measures sialidase activity of NA by detecting the terminal galactose that becomes exposed after sialic acid cleavage. A study conducted by Eichelberger et al^18^ that employed the protocol published by Couzens et al^17^ showed that the ELLA is robust and sensitive although improvements can be made to further standardization of the method.

Measuring the NI antibody only is possible if HA‐specific antibodies are unable to bind to the virus. This is usually accomplished by using reassortant viruses that have a mismatched avian HA, although it should be kept in mind that antibodies against conserved HA epitopes^19^ can still occur. The production of reassortant influenza viruses, beyond the intrinsic difficulty of optimizing the process, limits many laboratories from using these as a source of antigen since genetically modified organisms require additional biosafety containment and in some countries a permit from the Department of Agriculture. This is due to the HA gene, that is usually derived from an avian source. The inability of many laboratories to produce such reassortant viruses by reverse genetics have led to the employment of alternative sources of antigen. Jonges et al^20^ described Triton X‐100 treatment of wild‐type A/California/07/2009 (H1N1) X‐181 virus, which led to the disruption of virus particles while maintaining NA structure and activity. A simple and innovative solution has been investigated by Prevato et al^21^ and further characterized^22^; the employment of influenza lentiviral pseudotypes (pseudovirus or PV as will be called from now on) as a surrogate virus that expresses the human influenza NA (with or without an avian influenza HA) of interest. PV is reassortant chimera viruses that are infectious for a single cycle, thus unable to make infectious progeny. These characteristics allow many laboratories to handle them on lower biosafety containment levels. Triton X‐100 and PV as alternative antigens for the ELLA would avoid the need for reassortant viruses, so that ELLAs can be performed faster and safer. The employment of pseudovirus‐based ELLA assay (P‐ELLA) will also eliminate the need of wild‐type virus, meaning that this assay can be performed at biosafety level II (BSL2) even for influenza pandemic strains.

In this study, we compared three different types of antigens in the ELLA assay: reassortant viruses, Triton X‐100‐ inactivated viruses, and pseudoviruses. The comparison was performed by measuring NA inhibition antibody titers against N1 and N2 subtypes. Our results show correlation between titers measured with all the sources of antigens, providing the means to standardize and validate the methodology in laboratories that do not have access to suitable reassortant virus antigens.

## 2 MATERIALS AND METHODS

### 2.1 Reassortant viruses

H6N1 and H6N2 reassortant viruses expressing HA from A/turkey/Massachusetts/3740/1965 (H6N2), NA from A/California/07/2009 (H1N1) or A/Hong Kong/4801/2014 (H3N2) and the other genes from A/Puerto Rico/8/1934 (H1N1) were produced by reverse genetics as previously described.^17^ Reassortant viruses were inactivated by β‐propiolactone treatment.

### 2.2 Triton X‐100‐treated wild‐type viruses

Wild‐type A/California/07/2009 (H1N1) (code 13/198) and A/Hong Kong/4801/2014 (H3N2) (code 15/192) influenza viruses obtained from NIBSC were treated with different concentrations of Triton X‐100 (ranging from 0.1% to 1%) at 37°C for 1h as previously reported.^20^

### 2.3 Pseudoviruses

Production of lentiviral PVs was carried out by co‐transfecting HEK293T/17 (ATCC® CRL‐11268™) cells with phCMV1‐H11 (H11 from A/duck/Memphis/546/1974 (H11N9); kind gift of Davide Corti), pNLLuc4.3 (*gag‐pol* and *luc* genes, kind gift of Nathaniel Landau) and pI.18‐N1_Cal/09_ or pI.18‐N2_HK/14_ (backbone plasmid pI.18, kind gift of Carolyn Nicolson, NIBSC), as previously described.^22^, ^23^, ^24^, ^25^ The H11 plasmid was added to improve NA stability and increase the PV release and production as previously described^26^ and confirmed for this assay.^22^, ^25^ Briefly, 1 µg of HA, 1 µg of NA, and 1.5 µg pNLLuc4.3 plasmids were transfected into HEK293T/17 cell lines using Endofectin™ Lenti (3 µL/µg). Medium was replenished 24 hours after transfection. The NA activity of each PV was titrated in ELLA as reported previously.^17^

### 2.4 Serum samples

Two different panels of 40 (S1.1‐S1.40) and 34 (S2.1‐S2.34) human sera were tested against N1 and N2 NAs, respectively. Information about gender, age, and vaccination status was not provided.

### 2.5 HA assay

HA assays were performed to confirm the inability of Triton X‐100 treated virus to agglutinate RBCs. The protocol was described elsewhere (WHO 2011, Manual for the laboratory diagnosis and virological surveillance of influenza).

### 2.6 ELLA assay

ELLA assays were performed as previously described,^17^ with minor modifications. The amount of antigen used in the assay corresponded to 90% of the maximum signal. The quantity of horseradish peroxidase conjugated to peanut agglutinin from *Arachis hypogea* (HRPO‐PNA) corresponded to 1:1000 for all the tests except for the H11N2 PV, where a dilution of 1:500 was used. Titers were assigned as the 50% end point titer, that is, the inverse of the highest dilution that resulted in at least 50% inhibition of the maximum signal represented by the viral control (VC) minus the blank [(VC‐BLANK)/2)]. IC_50_ values were automatically generated using Graph Pad Prism 5® software.

### 2.7 Statistical evaluation

Every serum sample was tested in duplicate and evaluated by both 50% end point titer and IC_50_ outcomes.^22^ The percent inhibition of enzyme activity is calculated as follows: background optical density (OD) is subtracted from the virus control (maximum NA activity, no serum added) and sample ODs. The 50% end point titer is calculated as the highest serum dilution that resulted in at least 50% inhibition of the maximum NA activity. IC_50_ values were generated through a non‐linear regression curve fit using GraphPad Prism 5®, as described elsewhere.^25^

The geometric mean titer (GMT) of end point titers was reported as Log_2_ and compared by simple non‐linear regression curve fit, and *r* ^2^ (coefficient of determination)*,* a measure of strength of the relation between two variables.^27^ To investigate the relationship between the errors in measurement and the true values, the mean difference (*d*) and the standard deviations of the differences (*s*) were calculated.^27^ The Spearman rank calculation was also used to compare the outputs, as reported elsewhere.^28^

In addition, Log_2_ of IC_50_ titers determined from inhibitory curves were also compared. Coefficient of determination (*r^2^*), the mean difference (*d*), and the standard deviation of the differences (*s*) were evaluated by Bland and Altman and Spearman rank analyses.

## 3 RESULTS

### 3.1 Preparation of Triton X‐100‐treated antigens

Several concentrations of Triton X‐100 (Tx) were tested for their ability to selectively inactivate HA activity. Wild‐type A/California/07/2009 (H1N1) virus was completely inactivated by 0.5% and 1% Triton X‐100 treatment. Haemagglutination activity of wild‐type A/Hong Kong/4801/2014 (H3N2) was completely destroyed even by 0.1% Triton X‐100 concentration, as well as 0.5% and 1%. As in the method of Jonges et al,^20^ Triton X‐100 was not removed.

The impact of Tx‐treatment on NA activity was tested by titrating each preparation in ELLA. The activity of N1‐Tx preparations was slightly reduced compared to the untreated virus, but it did not impede test performance. To assess specificity of NA inhibition, the N1‐Tx wild‐type virus was pre‐incubated with anti‐A/California/7/2009 (N1) NA serum (NIBSC, code 10/218), anti‐A/California/7/09 HA serum (NIBSC, code 16/114), or human serum minus IgA/IgM/IgG (Sigma Aldrich cat. S5393‐1VL). The homologous antiserum against the HA did not show any inhibition of the NA. The immunoglobulin‐depleted human serum also did not inhibit NA. In contrast, the antiserum against NA inhibited enzyme activity of the N1‐Tx virus. The same titer was also obtained when the antiserum against NA was pre‐incubated with untreated wild‐type virus (results not reported).

Unfortunately, none of the N2‐Tx preparations retained NA activity. Even changing the buffer and the pH, as previously suggested,^29^ N2‐Tx preparations lost NA activity. Therefore, only N1‐Tx was included in the comparison of antigens to measure NA inhibition antibody titers.

### 3.2 H6N1, H1N1‐Tx, and H11N1 PV sources of antigen show comparable NI antibody titers

**Figure 1:**
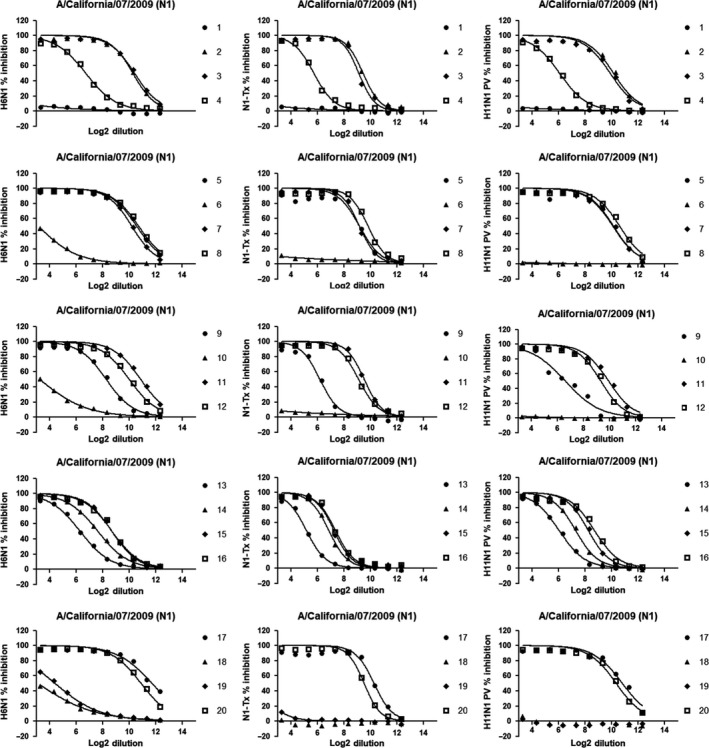
Inhibition curves showing the ability of sera to inhibit sialidase activity across the plate. Sera from 1 to 20 (corresponding to plates 1‐5) were tested in duplicate (GMTs here reported) against H6N1 reassortant virus (left column), N1‐Tx antigen (central column), and H11N1 PV (right column). Y axes represent the percentage of inhibition while X axes report the Log2 of the dilution. The prefix "S1." in front of every serum number is omitted to improve the readability of the legends

**Figure 2:**
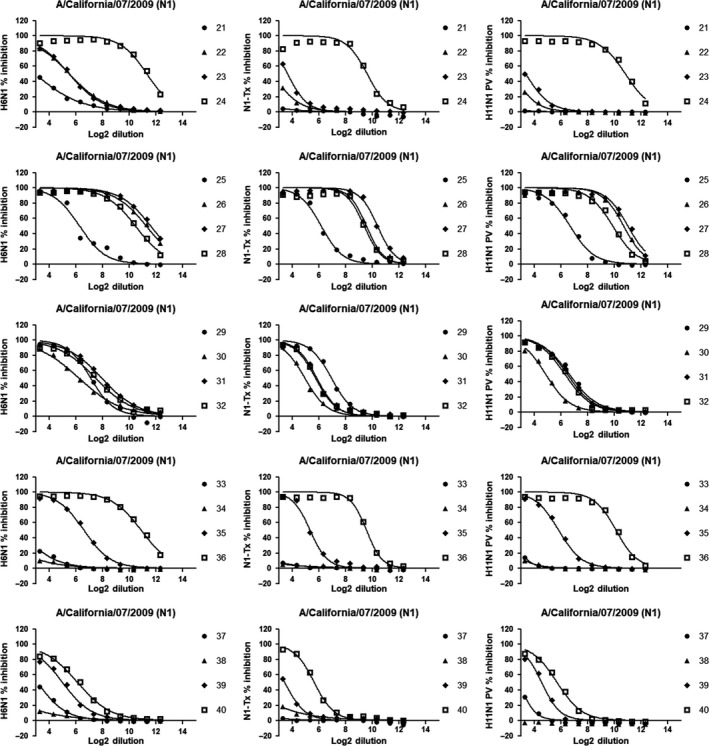
Inhibition curves showing the ability of sera to inhibit the sialidase activity across the plate. Sera from 21 to 40 (corresponding to plates 6‐10) were tested in duplicate (GMTs here reported) against H6N1 reassortant virus (left column), N1‐Tx antigen (central column), and H11N1 PV (right column). Y axes represent the percentage of inhibition while X axes report the Log2 of the dilution. The prefix "S1." in front of every serum number is omitted to improve the readability of the legends

H6N1 reassortant virus, N1‐Tx, and H11N1 PV were titrated in ELLA to determine the appropriate amount of antigen to use in each assay. The amount of antigen added to each assay was 90% of the maximum OD for each source of antigen. ELLA assays were performed to measure NI antibody titers of 40 sera against each N1 antigen, (Figures 1 and 2).

Table 1 shows that results are reproducible, with the NI titer of each replicate being within 2‐fold difference. To allow statistical comparison of results, titers measured as <10 (1:10 was the first dilution of serum) were assigned a titer of 5. In addition, Log_2_ of IC_50_ values, obtained using Graph Pad Prism 5®, were collected and analyzed.

**Table 1:** ELLA assay outcomes (S1.1‐S1.40) from the 3 different sources of antigen for N1 NA. Individual NI titers and GMTs of the results are shown

	Test 1	Test 2	Test 1	Test 2	Test 1	Test 2	AVG	AVG	AVG
Serum ID	N1‐TX	N1‐TX	H11N1‐PV	H11N1‐PV	H6N1	H6N1	N1‐TX	H11N1‐PV	H6N1
S1.1	5	5	5	5	5	5	5	5	5
S1.2	640	640	1280	1280	640	1280	640	1280	905
S1.3	320	320	640	640	1280	1280	320	640	1280
S1.4	40	40	40	40	80	80	40	40	80
S1.5	320	640	640	1280	1280	1280	453	905	1280
S1.6	5	5	5	5	5	5	5	5	5
S1.7	320	320	1280	640	640	640	320	905	640
S1.8	640	640	1280	1280	1280	1280	640	1280	1280
S1.9	40	80	80	80	320	320	57	80	320
S1.10	5	5	5	5	5	10	5	5	7
S1.11	640	640	640	640	1280	1280	640	640	1280
S1.12	320	320	640	640	640	640	320	640	640
S1.13	20	20	40	40	40	80	20	40	57
S1.14	80	80	160	160	160	160	80	160	160
S1.15	80	80	320	320	320	320	80	320	320
S1.16	80	80	320	320	320	320	80	320	320
S1.17	640	640	1280	1280	1280	2560	640	1280	1810
S1.18	5	5	5	5	5	5	5	5	5
S1.19	5	5	5	5	20	20	5	5	20
S1.20	640	640	1280	1280	1280	1280	640	1280	1280
S1.21	5	5	5	5	10	5	5	5	7
S1.22	5	5	5	5	40	40	5	5	40
S1.23	10	10	10	5	40	40	10	7	40
S1.24	640	640	1280	1280	2560	2560	640	1280	2560
S1.25	40	40	80	80	40	40	40	80	40
S1.26	640	640	1280	1280	2560	2560	640	1280	2560
S1.27	1280	640	1280	1280	2560	2560	905	1280	2560
S1.28	320	640	640	640	1280	1280	453	640	1280
S1.29	80	80	80	80	80	80	80	80	80
S1.30	20	20	20	20	40	40	20	20	40
S1.31	40	40	80	80	160	160	40	80	160
S1.32	40	40	80	80	160	160	40	80	160
S1.33	5	5	5	5	5	5	5	5	5
S1.34	5	5	5	5	5	5	5	5	5
S1.35	20	20	40	40	80	80	20	40	80

The inhibition curves were obtained by performing non‐linear regression from every serum run in duplicate (GMT is shown) against each N1 source of antigen (Figures 1 and 2).

N1‐Tx end point titers were similar to titers using H11N1 PV as antigen in 90% of all the cases (36/40) of which 17/36 were identical (42.5%). There was a greater than 2‐fold difference in only 4 cases. NI titers measured in assays using H11N1 PV and H6N1 reassortant viruses were similar in 90% of all the cases (36/40) of which 14 sera had the same titer (35%). There was a greater than 2‐fold and 4‐fold difference in only 2 cases each. Greater differences were found when N1‐Tx and H6N1 values where compared; 23/40 of all cases (57.5%) were well‐aligned and of those, 7/23 (17.5%) had the same titer. There was a greater than 2‐fold and 4‐fold difference in 17 and 1 cases, respectively.

In 7/40 (17.5%) of all the cases, the assays conducted with different antigen sources gave the same results, while 17/40 (42.5%) the results had 2‐fold difference and 14/40 (35%) had 4‐fold difference. Interestingly the titers obtained with N1‐Tx were approximately 2‐fold lower than the PV antigen, while titers obtained with H6N1 reassortant values were often 2‐fold higher than those measured against the PV antigen. Only in 1 case was an 8‐fold difference in titer observed between N1‐Tx or H11N1 PV and H6N1 (serum 22). In summary, titers from ELLA assays conducted with the three different sources of antigen varied not more than 4‐fold, except in one case.

**Figure 3:**
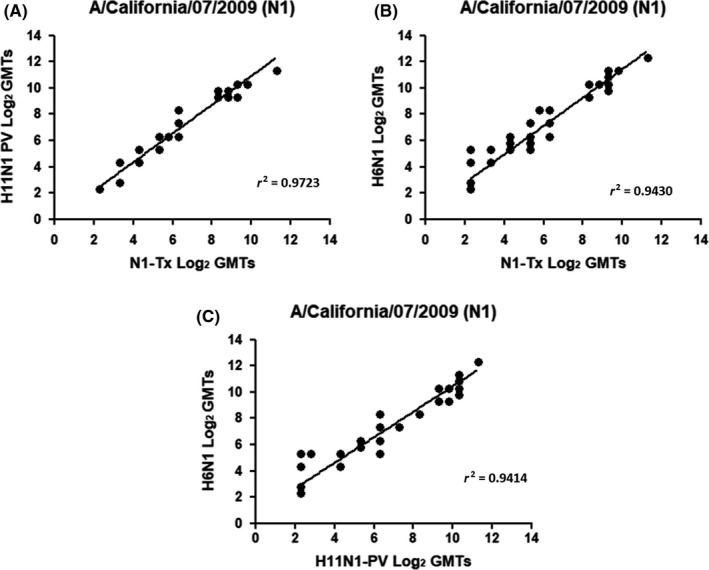
Comparison between (A) wild‐type N1‐Tx/H11N1 PV, (B) wild‐type N1‐Tx/H6N1, and (C) H6N1/H11N1 PV geometric end point titers. Results are represented as Log2 of the end point titer

A comparison of ELLA titers using three different sources of antigens was also performed (Figure 3).

**Figure 4:**
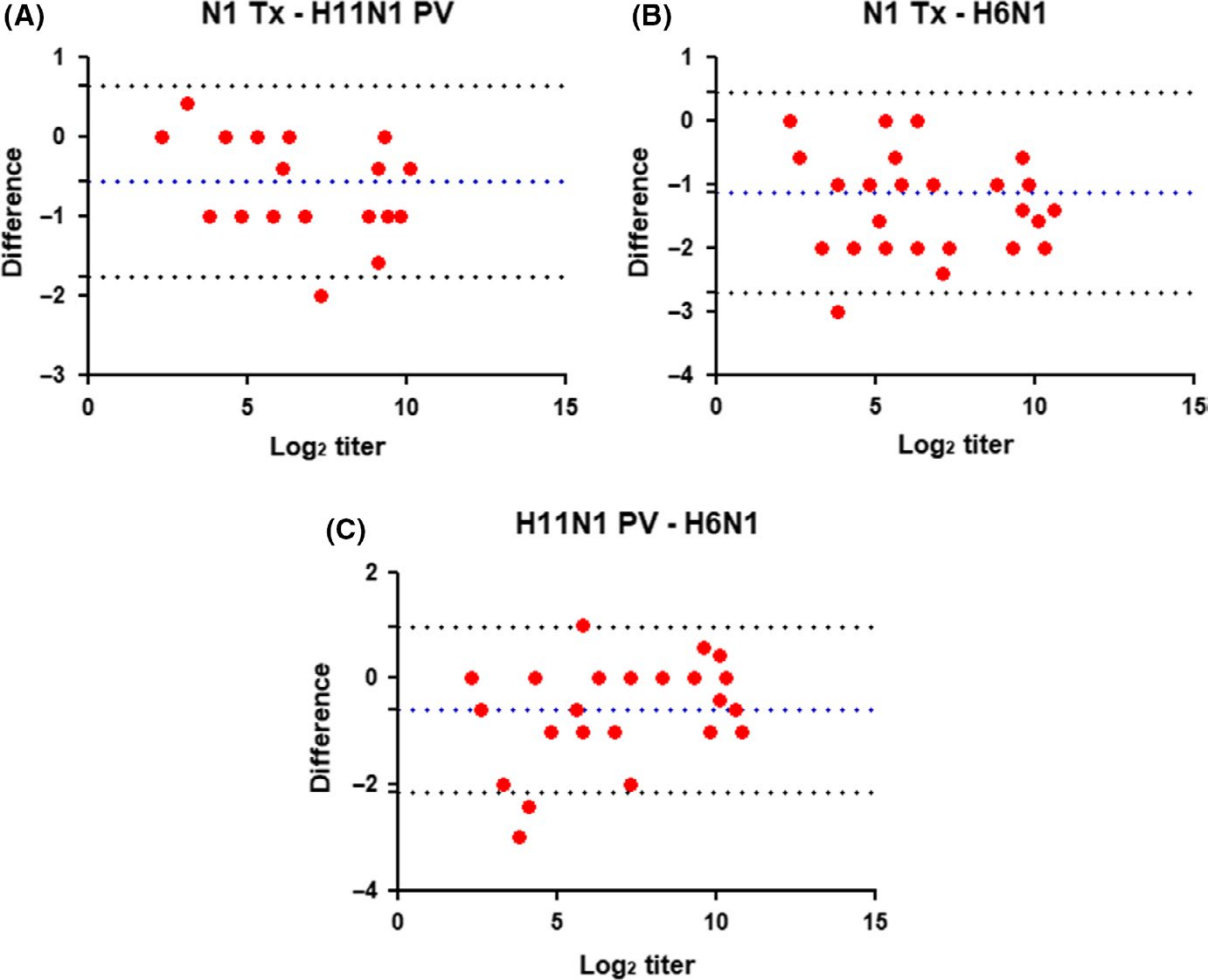
Bland‐Altman plot of the differences between (A) wild‐type N1‐Tx/H11N1 PV, (B) wild‐type N1‐Tx/H6N1, and (C) H11N1 PV/H6N1. The difference of titers (log 2) measured for each serum in ELLA using different sources of antigen (red bullets) plotted against the limit of agreement (±1.96 SD, stippled black line) and the overall mean of the different outcomes (bias, stippled dark blue line). There are no significant differences at high or low titers

Comparison between N1‐Tx and H11N1 PV (Figure 3A) yields an *r^2^* = 0.9723. Interestingly both these two sources of antigens are comparable to H6N1 (Figure 3B,C), yielding *r^2^* values of 0.9430 and 0.9414 with N1‐Tx and H11N1 PV, respectively. To assess, whether there was any bias Bland and Altman correlation analysis^27^ was performed (Figure 4).

This confirmed that the two measurements are comparable, with very few titers outside the intervals defined as “limits of agreement.” However, titers measured against N1‐Tx were often less than measured against H11N1 PV (Figure 4B), and titers measured against H6N1 were often higher than those measured by either N1‐Tx or H11N1 PV (Figure 4A,C, respectively). The difference in titers measured for sera 22 and 23 was outside the limits of agreement for assays using H6N1 and H11N1 PV (Figure 4C).

**Figure 5:**
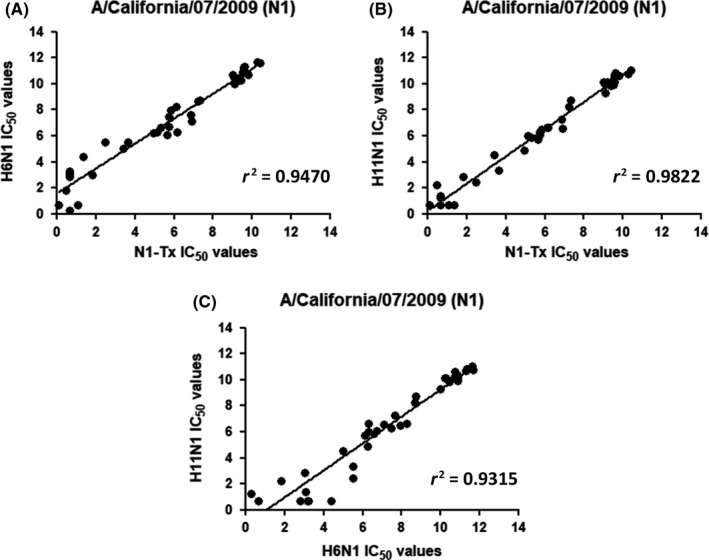
Comparison between (A) wild‐type N1‐Tx/H6N1, (B) wild‐type N1‐Tx/H11N1 PV, and (c) H6N1/H11N1 PV, IC50 titers. Results are represented as Log2 of the IC50 titers

IC_50_ titers were compared through linear regression (Figure 5). Comparison between N1‐Tx and H11N1 PV IC_50_ titers (Figure 5B) shows an *r^2^* = 0.9822, and an *r^2^* = 0.9470, and *r^2^* = 0.9315 when compared to H6N1 outcomes, respectively (Figure 5A,C).

**Figure 6:**
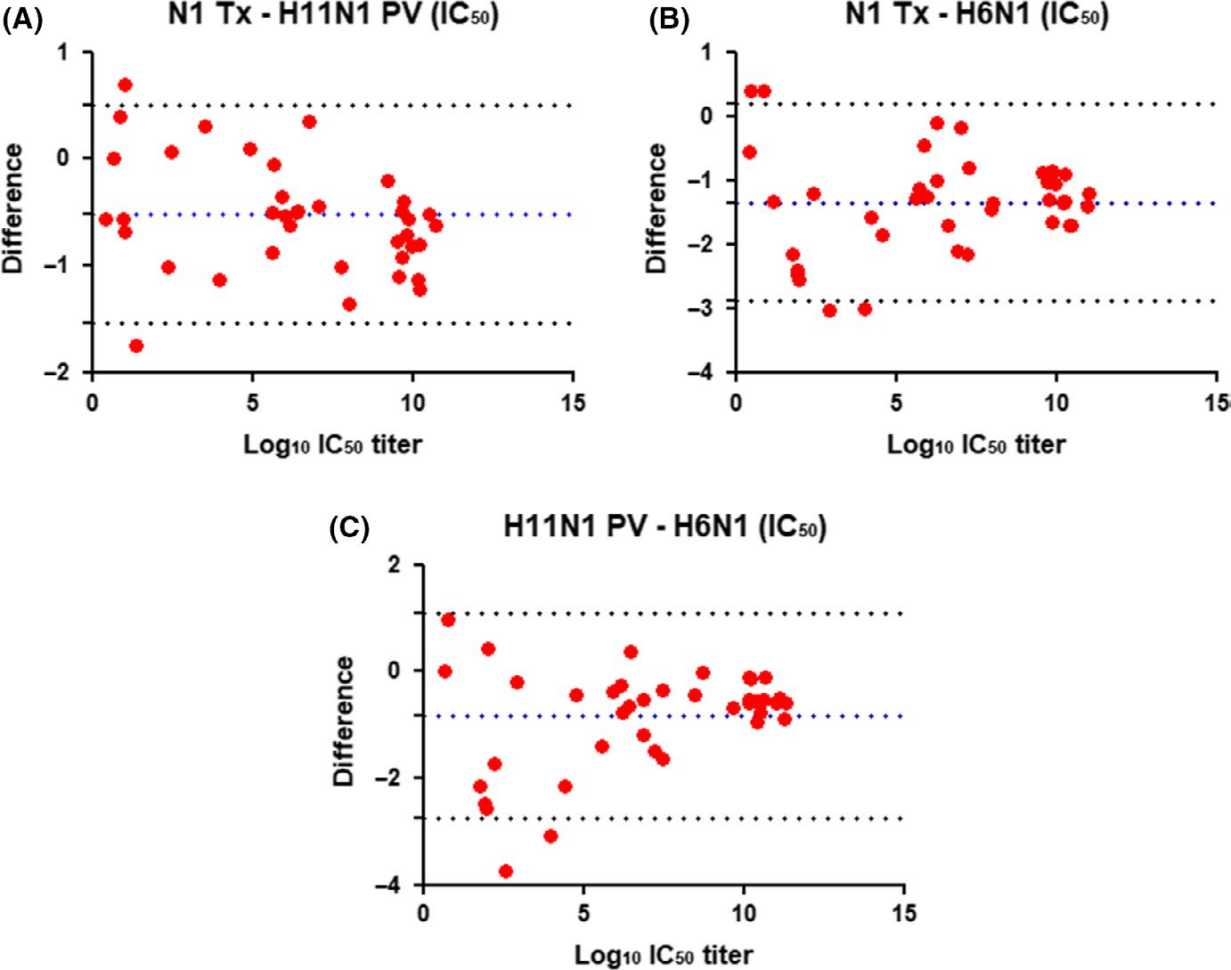
Bland‐Altman plot of the differences between (A) wild‐type N1‐Tx/H11N1 PV, (B) wild‐type N1‐Tx/H6N1 and (C) H11N1 PV/H6N1 (C), IC50s respectively. The mean difference of Log2 IC50 titers measured in assays using different antigens (red bullets) are plotted for each serum. The limit of agreement (±1.96 SD, stippled black line) and the overall mean of the differences (bias, stippled dark blue line) is shown on each graph

As expected, the Bland‐Altman analyses performed with 50% end point and IC_50_ titers are similar (Figures 4 and 6). The Bland‐Altman analysis of IC_50_ titer differences showed a trend for larger differences measured by the three assays at low titers (Figure 6). This is evident from the titers reported in Table 1; sera 19, 22, and 23 all have low or unmeasurable (<10) titers using PV and N1‐Tx antigens, but a reasonable titer measured in assays using H6N1 as antigen.

### 3.3 NA inhibition antibody titers measured in ELLA with H6N2 and H11N2 PV sources of antigen are similar

**Figure 7:**
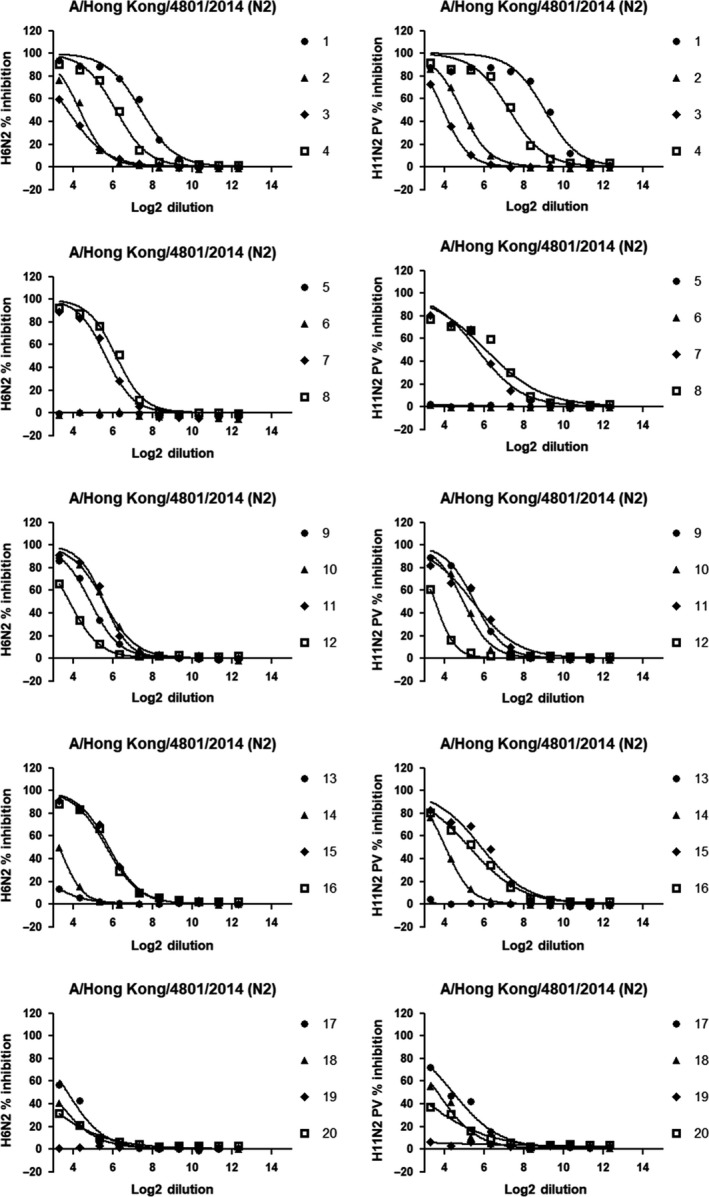
Inhibition curves showing the ability of sera to inhibit the sialidase activity across the plate. Sera from 1 to 20 (corresponding to plates 1‐5) were tested in duplicate (GMTs here reported) against H6N2 reassortant virus (left column) and H11N2 PV (right column). Y axes represent the percentage of inhibition while X axes report the Log2 of the dilution. The prefix "2." in front of every serum number is omitted to improve the readability of the legend

**Figure 8:**
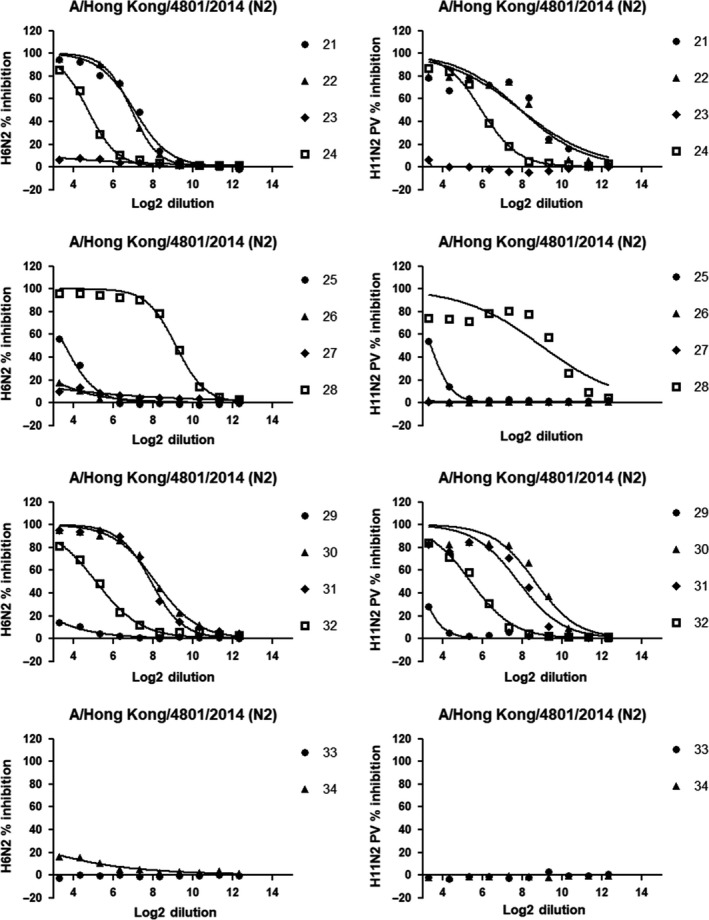
Inhibition curves showing the ability of sera to inhibit the sialidase activity across the plate. Sera from 21 to 34 (corresponding to plates 6‐9) were tested in duplicate (GMTs here reported) against H6N2 reassortant virus (left column) and H11N2 PV (right column). Y axes represent the percent inhibition while X axes report the Log2 of the serum dilution. The prefix "2." in front of every serum number is omitted to improve the readability of the legends

Since an N2‐Tx virus was not available, NI antibody titers measured by ELLA using only H6N2 and H11N2 PV antigens were compared (Figures 7 and 8).

The protocol used for each antigen was the same, with the exception of HRPO concentration which was used at a higher concentration (1:500) for H11N2 PV to improve the signal (internal results, not showed). Results were analyzed as described in the methods.

End point titers measured using H6N2 and H11N2 PV as antigens are similar (2‐fold or less difference) in 88% of cases (30/34), with 19 of 34 titers being identical (56%). Only 4 sera had a 4‐fold difference (12%) in 1 of 2 replicates. Differently from N1 results, there was excellent consistency in both high and low titer replicate measurements within each assay and between assays using different antigens. The end point titers measured in both assays are shown in Table 2.

**Table 2:** ELLA assay outcomes (S2.1‐S2.34) deriving from 2 different sources of antigens (N2 NA). Average of the results are also showed

	Test 1	Test 2	Test 1	Test 2	AVG	AVG
Serum ID	H11N2‐PV	H11N2‐PV	H6N2	H6N2	H11N2‐PV	H6N2
S2.1	320	640	160	160	453	160
S2.2	20	20	20	20	20	20
S2.3	10	10	10	10	10	10
S2.4	80	160	40	40	113	40
S2.5	5	5	5	5	5	5
S2.6	5	5	5	5	5	5
S2.7	40	40	40	40	40	40
S2.8	80	80	80	80	80	80
S2.9	40	40	20	20	40	20
S2.10	20	20	40	40	20	40
S2.11	40	40	40	40	40	40
S2.12	10	10	10	10	10	10
S2.13	5	5	5	5	5	5
S2.14	10	10	5	10	10	7
S2.15	80	40	40	40	57	40
S2.16	40	20	40	40	28	40
S2.17	10	20	10	10	14	10
S2.18	10	10	5	5	10	5
S2.19	5	5	5	5	5	5
S2.20	5	5	5	5	5	5
S2.21	320	320	80	160	320	113
S2.22	320	160	80	80	226	80
S2.23	5	5	5	5	5	5
S2.24	40	40	20	20	40	20
S2.25	10	10	10	10	10	10
S2.26	5	5	5	5	5	5
S2.27	5	5	5	5	5	5
S2.28	640	640	320	320	640	320
S2.29	5	5	5	5	5	5
S2.30	320	320	160	160	320	160
S2.31	160	160	160	160	160	160
S2.32	40	20	20	20	28	20
S2.33	5	5	5	5	5	5
S2.34	5	5	5	5	5	5
HP‐HS‐N2	1280	1280	2560	2560	1280	2560
Minus	5	5	5	5	5	5

**Figure 9:**
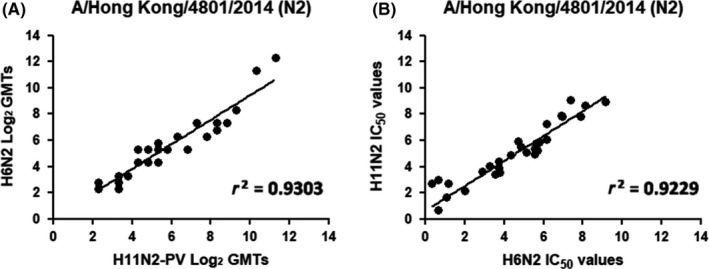
Comparison of NI titers measured in ELLA using H6N2 and H11N2PV antigens. Results are shown as (A) Log2 of the end point titer and (B) IC50 titers

The correlation between the H6N2 and H11N2 PV end point and IC_50_ titers (Figure 9) shows a good concordance of results, with correlation coefficient of *r^2^* = 0.9303 and *r^2^* = 0.9229, respectively. Interestingly the N2‐specific GMTs measured using these different antigens are closer than N1‐specific GMTs measured in ELLA using H11N1 PV and H6N1 antigens. Nevertheless, the higher variety in titers within the N2 panel has probably determined a lesser consistency between data, affecting the coefficient of correlation.

**Figure 10:**
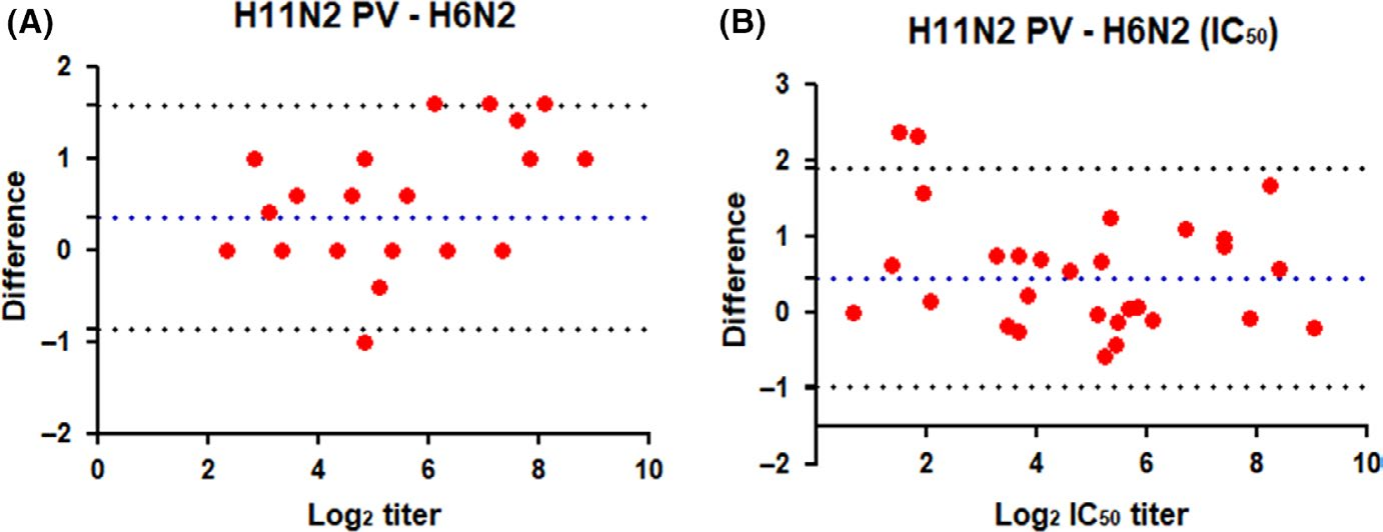
Bland‐Altman plot of the differences between (A) H6N2/H11N2 PV Log2 end point and (B) IC50 titers. The mean difference of each value (red bullets) between the ELLA run with different sources of antigen is plotted, with the limit of agreement (±1.96 SD, stippled black line) and the overall mean of differences (bias, stippled dark blue line) are shown

As for N1 titers, the Bland‐Altman analysis on N2 titers show small differences in titers measured using the different antigens when either end point titers or IC_50_ titers were plotted (Figure 10). Only one serum (10) was greater than the higher limit of agreement, when end point titers measured by H11N2 PV were compared with H6N2 titers. In fact, this is the only case in which the titer measured in an assay using H11N2 PV antigen is 2‐fold higher than the titer measured with H6N2 as antigen. In addition, sera 23 and 29 were greater than the higher limit of agreement, when differences in IC_50_ titers from assays using H11N2 PV and H6N1 antigens were compared. Both sera 23 and 29 had no measurable NI antibodies (titer of 5) to inhibit H11N2 PV and H6N2 antigens. This explains why there is no difference between the 50% end point titers. However, it impacts the IC50 titer because it is not possible to establish a lower limit of quantitation (LLOQ) from which to determine an accurate IC_50_ value. The problem of determining an IC50 for samples that have low antibody titers is evident in viewing the H11N2 PV curves for sera 23 and 29 (Figure 8).

The correlations of titers were also evaluated by Spearman rank analysis for both N1 and N2 assays. As shown in Table 3, all the results showed good correlation when either IC_50_ or end point titers were evaluated.

**Table 3:** Correlation coefficients of Spearman rank and regression analyses of end‐point and IC50 titres measured assays using different antigen sources

Antigen	Spearman rank		Linear regression method	
	End point	IC50	End point	IC50
H6N1/H11N1	0.9586	0.9665	0.9414	0.9315
H6N1/N1‐Tx	0.9631	0.9750	0.9430	0.9470
H11N1/N1‐Tx	0.9822	0.9783	0.9723	0.9822
H6N2/H11N2	0.9668	0.9569	0.9303	0.9229

In conclusion, measurement of NI antibody titers against N1 and N2 antigens by ELLA demonstrate comparable results using PV or Triton X‐100‐disrupted virions and the gold‐standard H6 reassortant viruses.

## 4 DISCUSSION

This is the first study that compares NI antibody titers from assays employing three different sources of NA. The study included panels of sera tested against N1 and N2 antigens in the form of whole influenza virus and lentivirus pseudotype viruses with mismatched HAs, as well as Triton X‐100‐disrupted wild‐type H1N1 influenza virus. A previous study was conducted comparing reassortant mismatched viruses and PV as sources of antigen,^22^ but due to the different subgroups of NA used, a direct comparison of antigen equivalence was not possible at that time.

Treatment of N2 with Triton X‐100 was not optimal and unfortunately did not result in enzymatic activity; therefore, this antigen could not be included in the study. Wild‐type virus A/Hong Kong/4801/2014 (H3N2) was treated to different Tx concentrations, at different temperatures and at different incubation times, but all of them resulted in no NA activity. Additional investigations need to identify optimal conditions for Triton X‐100 treatment of influenza viruses that retain NA activity. This may include evaluating calcium concentration and a wider pH ranges in buffer and further detergents.

Each serum (panel N1 or N2) was run twice within each plate (one reportable value generated) against one source of antigen per day, in two different days. The raw data obtained were evaluated as end point titers and IC_50_ titers. The linear regression, Spearman rank, and Bland‐Altman analyses were employed to assess the comparability of results. The correlation coefficients for all comparisons were high (Table 3), suggesting the suitability of performing ELLA with any of these antigen sources. As observed from the Bland‐Altman analyses, titers measured against N2 antigens were in better agreement than N1 titers measured using H6N1 and H11N1 PV antigens. This suggests the N1 assays did not have equivalent sensitivity. This may be a result of using different absolute amounts of NA in each assay or the impact of extrinsic factors on assay performance. Further studies are needed to examine the reason for higher titers being measured using H6N1 as the antigen. In fact, it can either depend on different antigen accessibility of antibodies or different sensitivity of the assay.^19^ Although a 4‐fold increase is usually considered seroconversion when pre‐ and post‐vaccination sera are evaluated according to international harmonization guidelines, these assay still need to be validated to draw more robust conclusions. Multiple runs with the same panels of sera should ensure stronger reproducibility data in support of it.

Lentiviral PVs are a chimeric surrogate for influenza virus that can be employed to assess antibodies against NA without safety concerns. Even though avian HAs (ie, H11, but other non‐human HA can be co‐expressed) are expressed on its surface, there is very low risk of infection to avian species or humans. The HA subtype can easily be switched to co‐express a different avian HA with human NA if needed, providing a practical means to perform assays without the need for generating reassortant influenza viruses that need to be handled more stringently.^21^, ^25^ A previous comparison between NI titers using H11N1 and N1 only PVs in ELLA highlighted differences in titers.^22^ Whether this was due to PV stability, HA serum inhibition, or interference by antibodies that bind HA stem^19^ is unclear and should be further investigated.

Additional analysis on NA content and activity of the three sources of antigens would help optimize their use in ELLA. For example, scanning electron microscopy (SEM) or transmission electron microscopy (TEM) would better characterize the sources of antigen and their structure. Beside this, further comparisons to evaluate the applicability of our findings to other strains and subtypes of NAs would strengthen the results of our study, particularly if performed in multiple laboratories.

In conclusion, NA inhibition antibody titers measured in ELLA performed with three different sources of antigen are similar and suggest lentiviral PV can be used to evaluate anti‐neuraminidase responses. Further analyses with additional N1 and N2 strains and subtypes to strengthen this finding will be of value.
